# Lethal *Clostridium difficile* Colitis Associated with Paclitaxel and Carboplatin Chemotherapy in Ovarian Carcinoma: Case Report and Review of the Literature

**DOI:** 10.1155/2010/749789

**Published:** 2010-07-18

**Authors:** V. Masciullo, S. Mainenti, D. Lorusso, P. A. Margariti, G. Scambia

**Affiliations:** Division of Gynecologic Oncology, Catholic University of Sacred Heart, L. go A. Gemelli, 8, 00168 Rome, Italy

## Abstract

Clostridium difficile colitis, although rare, could represent a serious complication following chemotherapy. Prior antibiotic use has been considered the single most important risk factor in the development of *C. difficile* infection. Recently, the association between antineoplastic therapy and *C. difficile*-associated diarrhea in the absence of a prior antibiotic therapy has become more apparent. A 75-year-old woman with serous adenocarcinoma of the ovary developed lethal pancolitis caused by *C. difficile* after five cycles of paclitaxel- and carboplatin-based chemotherapy. She presented with diarrhea, coffee-ground emesis, and oliguria and was hospitalized immediately for aggressive treatment. Despite all the medical efforts, her condition worsened and she died after twenty days. We describe the second case reported of a patient developing a severe *C. difficile* colitis following chemotherapy without any recent antibiotic use and review the data of the literature, emphasizing the need to a prompt diagnosis and management that can significantly decrease the morbidity and life-threatening complications associated with this infection.

## 1. Introduction

Combination chemotherapy regimens including paclitaxel have been widely used for standard treatment of many solid tumors, including ovarian cancer [1]. Recently, a range of acute serious gastrointestinal complications such as necrotizing colonic inflammatory disorders, pancolitis, and ischemic colitis have been reported in paclitaxel containing regimens [2–6].

 The association between cancer chemotherapy and *Clostridium difficile*-associated colitis has recently become more apparent; however, the exact mechanism is poorly understood. Prior antibiotic therapy is considered the single most important risk factor in the development of *C. difficile*-associated diarrhea. Nevertheless, few cases of patients affected by severe *C. difficile* colitis following chemotherapy in the absence of recent antibiotic use have recently been reported [7, 8].

Here, we describe a case of a woman with advanced ovarian carcinoma who developed severe pancolitis caused by *C. difficile* after five cycles of paclitaxel and carboplatin chemotherapy without any recent antibiotic use. Moreover, we reviewed the literature concerning the management and treatment of patients with severe *C. difficile* infection.

## 2. Case Report

In September 2007, a 75-year-old Caucasian woman with stage IIIB papillary serous ovarian adenocarcinoma was admitted in the Division of Gynecologic Oncology of the Catholic University. She underwent an explorative laparoscopy, for a pelvic mass, diffuse peritoneal carcinomatosis, and ascites. Within two weeks the patient received the first cycle of Paclitaxel (135 mg/m^2^) and Carboplatin (AUC 4). The first four cycles were well tolerated, showing no major hematologic or organ toxicity with complete response to chemotherapy. Therefore, she underwent optimal cytoreductive surgery with no residual disease left in the pelvis and the upper abdomen. One month after surgery, the patient received her fifth cycle of chemotherapy. The day after the administration of chemotherapy, she reported abdominal pain, fever (101, 3°F), and asthenia, and after 4 days. She presented diarrhea, coffee-ground emesis, and oligoanuria. 

**Figure 1:**
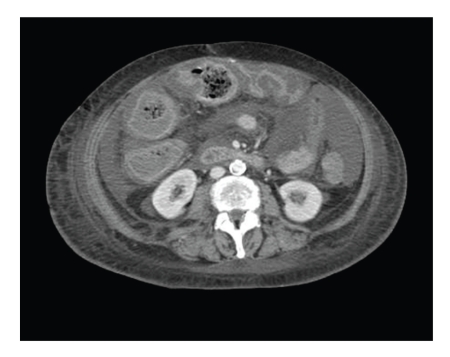
A CT-scan of the abdomen showing increased thickness of the colonic wall.

Hematologic laboratory values were haemoglobin (g/dl) 8, 9, white blood cell count 4.70 × 10^9^/l. Physical examination revealed a diffuse abdominal tenderness, distension, and guarding. A CT-scan (Figure 1) revealed an increased thickness of colonic wall compatible with diffuse severe pancolitis. White blood cell count was 1.39 × 10^9^/l. Patient's blood cultures were negative. Assay of stool specimens revealed Clostridium difficile toxin. The patient's sepsis was treated with intravenous metronidazole (500 gr i.v, 4 times a day), *β*-lactamic antibiotic (1 gr i.v, 3 times a day), and teicoplanin (600 mg i.v). Oral vancomycin (500 mg 4 times a day) was also administered by nasogastric tube. The patient's condition worsened, and after 7 days of therapy she was transferred in the Intensive Care Unit of our hospital.

At a day twenty following admission for chemotherapy, she died.

## 3. Discussion

The mechanism of colitis as a complication of taxane-based chemotherapy is still unknown [9, 10]. Paclitaxel, as well as docetaxel, binds to the *β* subunit of tubulin, which results in the formation of stable, nonfunctional microtubule bundles that interfere with mitosis [11, 12]. 

*C. difficile* is an etiologic agent for antibiotic-associated diarrhea (15%–25% of all cases) and pseudomembranous colitis (95%–100% of all cases) [13]. Prior antibiotic therapy (in particular broad-spectrum antibiotics with activity against enteric bacteria, such as clindamycin, but also penicillins and cephalosporins) is considered the single most important risk factor in the development of *C*. *difficile*-associated diarrhea. However, the association between antineoplastic therapy and *C. difficile* infection in the absence of a prior antibiotic therapy has recently become more apparent [13]. Among patients with gynaecologic cancer and diarrhea without associated antibiotic use, the incidence of *C. difficile* has been found to be 8% in ovarian cancer patients [14], 6% in cisplatin-based chemotherapy [15], 2,2% in patients receiving standard-dosepaclitaxel containing regimens and as high as 20% in those treated with high-dose regimens [16]. Carboplatin has been reported to have less intestinal mucosal toxicity than cisplatin [17], and to our knowledge, only one case of *C. difficile* colitis induced by carboplatin used in association with paclitaxel has been previously reported [8]. The severity of *C. difficile* infection ranges from an asymptomatic carrier status to life-threatening pancolitis. Most commonly, *C. difficile* colitis presents as mild to moderate diarrhea associated with occasional abdominal cramps. As the severity of the disease increases, systemic manifestations include fever, leucocytosis, nausea, dehydration associated with profuse diarrhea, and abdominal pain and distension. Plain abdominal radiographs are usually not specific, and the diagnosis is usually confirmed by CT scan and, as the inflammatory process in the colon may be localized or diffuse, CT would be also useful in assessing the extent of the colitis [18].

For patients with severe or fulminant infection whose gastrointestinal tracts are functioning, oral vancomycin is the preferred therapy [19]. A recent double-blind randomized cinical study by Zar et al. indicated that the clinical cure rate for vancomycin in patients with severe CDI is significantly better than for metronidazole (97% versus 76%; *P* < .02) [19].

Whereas, the best treatment for patients with compromised gastrointestinal tract function remains controversial [18–26]. For these patients, delivery of reliable concentrations of orally administered drug to the site of infection cannot be assured. Some experiences support the use of intravenous metronidazole for treatment of *C. difficile* diarrhea [20, 21]. However, alternative methods to ensure effective antimicrobial concentrations at the site of infection should also be undertaken. For example, oral vancomycin should be given in addition to intravenous metronidazole. When severe adynamic ileus is suspected, intraluminal vancomycin should be considered, by a long catheter in the small intestine [22], direct intracolonic instillation [23], or rectal delivery through an enema [23–25]. If these approaches are unsuccessful and the patient's clinical condition deteriorates, subtotal colectomy with a temporary diverting ileostomy is the only life-saving alternative [26].

The occurrence of *C. difficile* infection in patients undergoing chemotherapy for gynaecological cancer is not rare and should be considered and treated promptly in the differential diagnosis of patients presenting diarrhea. Although the incidence of *C. difficile* infection is more frequent in those patients treated simultaneously with antibiotics and chemotherapeutic agents, this is the second case reported of a patient developing a severe *C. difficile* colitis following paclitaxel and carboplatin regimen without any recent antibiotic use [8]. Aggressive supportive care with i.v. hydratation, broad-spectrum antibiotics, and close surgical monitoring for selective intervention can significantly decrease the morbidity and life-threatening complications associated with this infection.
